# Travel risk behaviours and uptake of pre-travel health preventions by university students in Australia

**DOI:** 10.1186/1471-2334-12-43

**Published:** 2012-02-17

**Authors:** Anita E Heywood, Meng Zhang, C Raina MacIntyre, Holly Seale

**Affiliations:** 1 School of Public Health and Community Medicine, Faculty of Medicine, University of New South Wales, Kensington, New South Wales, Australia; 2 Faculty of Medicine, University of New South Wales, Kensington, New South Wales, Australia; 3 National Centre for Immunisation Research and Surveillance of Vaccine Preventable Diseases (NCIRS), The Children's Hospital at Westmead and Discipline of Paediatrics and Child Health, University of Sydney, Westmead, New South Wales, Australia

## Abstract

**Background:**

Forward planning and preventative measures before travelling can significantly reduce the risk of many vaccine preventable travel-related infectious diseases. Higher education students may be at an increased risk of importing infectious disease as many undertake multiple visits to regions with higher infectious disease endemicity. Little is known about the health behaviours of domestic or international university students, particularly students from low resource countries who travel to high-resource countries for education. This study aimed to assess travel-associated health risks and preventative behaviours in a sample of both domestic and international university students in Australia.

**Methods:**

In 2010, a 28 item self-administered online survey was distributed to students enrolled at the University of New South Wales, Sydney, Australia. Multiple methods of distributing links to the online survey were utilised. The survey examined the international travel history, travel intentions, infection control behaviours and self-reported vaccination history.

**Results:**

A total of 1663 respondents completed the online survey, 22.1% were international students and 83.9% were enrolled at an undergraduate level. Half had travelled internationally in the previous 12 months, with 69% of those travelling only once during that time with no difference in travel from Australia between domestic and international students (*p *= 0.8). Uptake of pre-travel health advice was low overall with 68% of respondents reporting they had not sought any advice from a health professional prior to their last international trip. Domestic students were more likely to report uptake of a range of preventative travel health measures compared to international students, including diarrhoeal medication, insect repellent, food avoidance and condoms (*P *< 0.0001). Overall, students reported low risk perception of travel threats and a low corresponding concern for these threats.

**Conclusions:**

Our study highlights the need to educate students about the risk associated with travel and improve preventative health-seeking and uptake of precautionary health measures in this highly mobile young adult population. Although immunisation is not an entry requirement to study at Universities in Australia, large tertiary institutions provide an opportunity to engage with young adults on the importance of travel health and provision of vaccines required for travel, including missed childhood vaccines.

## Background

The international education sector has continued to evolve enormously in the last two decades. In 1990, Australia welcomed 47,000 international students and by 2000, this number had grown to 188,000 [1]. In 2010, there were 469,619 enrolments by full-fee paying international students on student visas, with 227,230 (48%) enrolled in higher education courses [2]. International students originate from more than 190 countries-from major cities to remote rural villages, primarily from China and India, with approximately half aged 20-24 years and a quarter aged 25-29 years [2]. Entrants to Australia on student visas, including international university students, comprise a increasing proportion of travellers, from 3.7% of short-term arrivals in 2000 to 6.3% in 2010 [3].

All entrants to Australia have the potential to import disease, including visitors and returning Australians. There are limited data on the infectious disease risks for international students from low resource countries studying in high resource countries, such as Australia. However, recent examples highlight the need to understand this globally mobile group. In 2007, a case of poliomyelitis in an international student returning to Australia from a short trip to Pakistan to visit friends and relatives was the first case in Australia since 1977 [4]. A 10 year review of tuberculosis (TB) cases in the state of Victoria, Australia found 37.9% of multi-drug resistant TB patients were international students [5]. Domestic university students may also be at an increased risk of importing infectious diseases as a result of missed childhood vaccinations during periods of declining disease transmission combined with high rates of travel to areas of high infectious disease endemicity. Currently in Australia, childhood immunisation coverage is high, with > 90% of children aged 24 months assessed as fully immunised and close to 90% of 6 year olds [6]. However, coverage at 24 months during the 1990s, was well below 75%, [6] and young Australian adults entering University, may well be susceptible to a number of vaccine preventable diseases. In Australia, young adults returning from abroad have been implicated in a number of measles clusters in the past decade, including one outbreak involving 74 cases [7]. Furthermore, the congregation of students with high rates of social contact may amplify infectious disease outbreaks. The importation of mumps by a student to a university in Iowa, USA in 2006 resulted in a large sustained outbreak, both within the student campus and outside the student community, with waning immunity in this population group implicated as a contributing factor [8].

Prior to and during travel abroad, travellers often do not seek or follow appropriate travel health advice from travel health professionals [9]. Of travellers who do not seek pre-travel preventative health advice, many may not be aware of the health risks and the protection available from pre-travel immunisation. Of those who do seek advice, a substantial proportion of travellers utilize information sources unlikely to provide sufficient, tailored information on risks, preventative measures and vaccinations such as travel agents, the internet or family and friends [10]. As a result, many travellers undertake their journey unprepared and susceptible to potential infectious disease threats [10]. Forward planning and preventative measures before travelling can significantly reduce the risk of many vaccine preventable diseases such as measles and hepatitis A [11]. However, many travellers do not take advantage of available precautions which may be related to their knowledge, attitudes and practices towards infectious disease prevention.

In contrast to the USA, where many States and individual tertiary institutions require proof of vaccination prior to college enrolment, [12] there are no existing requirements at universities in Australia regarding proof of immunisation. International student visa applications require x-ray screening for tuberculosis if they are from medium or high risk TB countries. However, no immunisation records are reviewed upon entry to Australia or to University. International students are of particular interest in the study of travel-related infectious disease risks as many undertake multiple return visits to their home country during the course of their studies and they fall outside the health systems which target resident and migrant populations. Because of the continued threat of importation and local spread of vaccine-preventable diseases, vaccination of this at-risk group should be a high priority. Although there have been several studies on Australian travellers in general, there is still limited information on the uptake of vaccination and preventative health advice in susceptible populations such as young domestic or international students studying in Australia. This study aimed to supplement this paucity of research related to the travel health risk appraisal, pre-travel health advice-seeking behaviours and vaccination uptake of young university students in Australia.

## Methods

### Participants

An anonymous online cross-sectional survey was conducted among students of the University of New South Wales (UNSW). All students enrolled during the study period were eligible to participate, regardless of their course, year of enrolment or stage of candidature. Recruitment of participants was from the study population of approximately 46,302 students, including 11,686 international students based on enrolments for the previous calendar year (2009) at UNSW. The anonymous online survey was placed on a host survey website "SurveyMonkey" (SurveyMonkey.com, LLC, USA) and the link was made available to all students from the University of New South Wales website between July 23^rd ^2010 and September 22^nd ^2010.

Multiple methods were employed to raise awareness of the survey. All Head of Schools from all Faculties were informed of the study via email and requested to forward survey information and the link to student coordinators and student representatives. The link for the survey was publicised through the School of Public Health and Community Medicine and Faculty of Medicine websites and via the main UNSW online student gateway. The study was further publicised through flyer handouts and posters on campus. Students were informed that the study was looking at risk perceptions and preventative practices towards infectious diseases and other threats, but the link to travel was not revealed. Students were incentivised to participate with a prize of small gift vouchers to four randomly selected eligible students who completed the survey. Ethics approval was provided through the Medical and Community Health Research Ethics Advisory Panel, University of New South Wales.

### Survey

A structured, 28 item self-administered questionnaire was designed with a primary focus on risk perception and identifying the travel practices of students. The questionnaire was developed through a literature review of previous survey-based research of international traveller behaviour, and the adaption of questions from previously published research carried out by study authors [13] and with permission from Hartjes et al [14]. Questions primarily focused on four vaccine-preventable diseases; hepatitis A, hepatitis B, influenza and measles. A pilot study was conducted on four students to assess accessibility, comprehension and relevance, with no necessary modifications identified.

The questionnaire contained the following: Eight items measured the participants travel history including the number of trips taken in the last 12 months and details of the most recent trip including travel destinations, length of stay, reasons for travel, and whether the respondent had sought travel health advice from any sources prior to departure. Several questions within this section were drawn from previously validated surveys [15]. Three items assessed the participant's knowledge of vaccine-preventable diseases, including examination of the participant's awareness of the availability of vaccines for some common travel diseases and a self-reported history of uptake of common travel vaccines prior to travel and health precautions used during travel in the past 12 months. Students were asked to select those diseases which were vaccine-preventable from a list of common travel diseases with several diseases with no vaccine available such as the common cold, HIV and dengue included as false responses.

Risk perceptions towards 13 different events were assessed using four items. In the first two items, participants were asked to indicate how likely they thought each event may occur for them while travelling internationally and while remaining in Australia. Likelihood was assessed on a 5-point scale (1 = not at all likely to 5 = very likely). In the second two items, participants were asked to indicate how worried they were that the 13 events would occur while in Australia and while travelling. Worry was assessed on a 5-point scale (1 = not at all worried to 5 = very worried). These risk perception questions were adapted with permission from previously validated questions developed by Hartjes et al [14], whose study was aimed towards a population group of US study abroad students. An additional question assessed perceptions of the seriousness of hepatitis A, dengue, measles and influenza infections. A 7-point scale (1 = extremely unaffected to 7 = extremely affected) was used to rate how students perceived how each of the four diseases would affect their health. Lastly, nine demographic items including age, gender, living arrangements, country of birth, stage of study, Faculty, time spent in Australia and country of residence concluded the survey.

### Analysis

Data were analysed using SPSS (16.0) for Windows, Rel. 11.0.1. 2001. Chicago: SPSS Inc and EpiInfo (version 3.3.2) CDC, Atlanta. Statistical association between categorical variables were analysed using Pearson chi-square tests and a *p*-value of < 0.05 was considered significant. Linear variables were analysed using independent and paired sample t-tests. During analysis, response categories such as the "effect of measles on health" were collapsed into "extremely affected" and "other" for the calculation of odds ratios.

## Results

### Participants

A total of 2,049 students responded to the online survey, of which 1,663 respondents (81%) completed all survey questions and were included in the final data. Based on total student population in 2009, approximately 4.4% (2,049/46,302) of students responded to the survey. Of students enrolled at UNSW during 2009, 25% were international students, compared to 22.1% in our survey population. Survey respondents were also younger (84.3% aged ≤ 24 years) overall than the UNSW student body in 2009 (67% aged ≤ 24 years). Of the 1,296 domestic students who completed a survey, 915 (70.6%) were Australian-born, with 54 other countries of birth represented. Of the international students (n = 367), 285 (77.7%) were from countries in Asia and most had been living in Australia for less than 2 years (n = 244, 66.5%). Other demographic characteristics, by enrolment status are shown in Table 1.

**Table 1:** Demographic characteristics and previous travel experience of University students respondents by enrolment status (N = 1663)

Variable	Domestic N (%)	International N (%)	*P *value*
Total participants	1296 (77.9)	367 (22.1)	-

**Demographics**			

***Study stage***			
Undergraduate	1161 (89.6)	234 (63.8)	< 0.001
Postgraduate	135 (10.4)	133 (36.2)	
***Age (years)***			
Mean (SD)	21.6 (5.2)	22.6 (3.3)	< 0.001^†^
***Gender*** Male	578 (44.6)	203 (55.3)	< 0.001
Female	718 (55.4)	164 (44.7)	
***Country of Birth***			
Australia	915 (70.6)	1 (0.3)	< 0.001
China (including Hong Kong)	116 (9.0)	157 (42.8)	
India	26 (2.0)	14 (3.8)	
Malaysia	16 (1.2)	36 (9.8)	
Other	223 (17.2)	159 (43.3)	

**Travel history**			

***Travelled in the past 12 months***	644 (49.7%)	185 (50.4%)	0.8
***Destination regions (past 12 months) ^‡§^***	*(N = 644)*	*(N = 185)*	
North East Asia	234 (36.3)	99 (53.5)	
South East Asia	208 (32.3)	72 (38.9)	
Europe	155 (24.1)	14 (7.6)	
Pacific	135(21.0)	22 (11.9)	
North America	92 (14.3)	12 (6.5)	
Middle East	39 (6.1)	6 (3.2)	
Africa	36 (5.6)	3 (1.6)	
South and Central America	26 (4.0)	3 (1.6)	
***Reason for travel (most recent trip) ^‡^***			
Holiday	358 (55.6)	78 (41.9)	< 0.001
Visit friends, relatives or return home	195 (30.3)	97 (52.2)	
Study Abroad	41 (6.4)	5 (2.7)	
Other^||^	50 (7.7)	6 (3.2)	
***Sought professional health advice (most recent trip) ^‡^***	246 (38.2)	23 (12.4)	< 0.001
*Source of advice:*			
General Practitioner/Family Doctor	228 (35.4)	16 (8.60)	< 0.001
Travel Clinic	14 (2.2)	2 (1.1)	0.5^¶^
University Health Service	15 (2.3)	9 (4.8)	0.07
Other Health Worker	2 (0.3)	0	-

* Pearson's Chi-squared test unless otherwise indicated

^† ^Two sample t-test

^‡^Includes those who had travelled internationally in the past 12 months only.

^§^May include more than one region

^|| ^Other includes employment, volunteering, attending conference/competition or for research (e.g. fieldwork/data collection)

^¶ ^Fisher's exact test

### Travel characteristics

Half (49.8%, 829/1,663) of all students reported that they had travelled to an international destination in the previous 12 months, of which 69.4% (578/829) reported one trip during this time. There was no significant difference in the proportion of domestic (49.7%) and international students (50.4%) who had travelled internationally in the last 12 months (*p *= 0.8). The most common travel destinations were in North and South East Asia (73.7%, 621/829) for both domestic and international students (Table 1). Of those travelling in the previous 12 months, 216 (26%) reported travel only to countries deemed low risk for infectious disease, including the USA, Canada, New Zealand, Japan and Europe. Significantly higher proportion of international students travelled outside these low risk destinations (170, 91.4%) compared to domestic students (444, 68.9%). Overall, travel for the purpose of a holiday was the most common reason cited for their most recent international trip (52.6%, 436/829). However, significantly more international students reported visiting friends and relatives compared to domestic students (52.2% vs. 30.3%, *p *< 0.001) (Table 1). Of the international students, 232 (63.2%) reported a return visit to their home country since commencing their current university course. 85 international students (23.2%) reported one visit and 147 (40.1%) reported two or more visits.

### Uptake of pre-travel health advice and preventative measures

Of the students who had travelled in the previous 12 months (n = 829), uptake of pre-travel health advice was low; with only 32.4% (269/829) of respondents reporting that they sought preventative health advice prior to their last trip from a health professional. Domestic students were significantly more likely to seek pre-travel health advice from a health professional (38.2% vs. 12.4% OR: 4.4, 95% CI 2.8-7.0; *P *< 0.001). Students aged 17-20 years (*P *= 0.04) and those who had only travelled once in the previous 12 months (*P *= 0.007) were significantly more likely to have sought pre-travel preventative health advice from a health professional prior to their last trip.

Of the students who indicated that they visited a health professional prior to their most recent trip, most reported seeing a general practitioner (90.7%, 244/269). Other sources of information are shown in Table 1. One fifth (20.9%, 173/829) reported also seeking information from other sources, with government websites such as the Smartraveller (a website of the Australian Federal Department of Foreign Affairs and Trade: http://www.smartraveller.gov.au/) the most commonly reported source. The second most common information source were family members or friends followed by travel guide books. Domestic participants who sought professional pre-travel preventative health advice were 2.4 times more likely to report seeking health information from other sources (31.7% vs. 16.1%, OR: 2.4, 95% CI: 1.7-3.5 *P *< 0.001). There was no association between seeking professional and other sources of health information for international students (*P *= 0.2). For domestic students, those who had travelled to visit friends or families were significantly less likely to seek health advice from a health professional (OR: 0.5, 95%CI: 0.4-0.7 *P *< 0.001) or other sources (OR: 0.5, 95%CI: 0.3-0.7 *P *= 0.001) than those travelling for other reason. For international students, there were no significant differences in pre-travel preventative health seeking by reason for travel (*P *= 0.4).

In this study, uptake of common vaccines was poorly reported. While a number of students reported ever receiving a vaccine for hepatitis B (36.4%), influenza (25.2%), hepatitis A (30.6%) and measles (20.9%), a substantial proportion of students were unable to recall if they had received a vaccine for hepatitis B (13.0%), influenza (13.2%), hepatitis A (16.4%) and measles (17.0%). Overall, 51.8% of students reported no or don't know for the four vaccines. Those who had sought professional pre-travel health advice were more likely to report a prior vaccination for hepatitis A (OR: 2.1, 95% CI: 1.5-2.9 *P *< 0.001), hepatitis B (OR: 1.9 95% CI: 1.4-2.5 *P *< 0.001) and influenza (OR: 2.3, 95% CI: 1.6-3.1 *P *< 0.001). Sun screen (567/829, 68.4%) and travel insurance (492, 59.3%) were stated as the two most common health precautions reported by students who had travelled in the past 12 months. Only 19.2% (192/829) of students reported carrying condoms with them on any trip in the past 12 months. Differences in uptake of precautionary travel health measures between domestic and international students are shown in Table 2.

**Table 2:** Uptake of travel health precautions for any international travel in the past 12 months by enrolment status (N = 829)

Health Precaution	All N = 829 (%)	Domestic N = 644 (%)	International N = 185(%)	*P*-value*	OR (95%CI)
Sunscreen	567 (68.4)	466 (72.4)	101 (54.6)	< 0.001	2.18 (1.6-3.1)
Travel insurance	492 (59.3)	425 (66.0)	67 (36.2)	< 0.001	3.42 (2.4-4.8)
Pain medication (e.g. paracetamol or aspirin)	476 (54.7)	408 (63.4)	68 (36.8)	< 0.001	2.98 (2.1-4.2)
Avoided certain foods/tap water	443 (53.4)	386 (59.9)	57 (30.8)	< 0.001	3.36 (2.4-4.8)
First aid kit	696 (49.1)	347 (53.9)	60 (32.4)	< 0.001	2.43 (1.7-3.4)
Insect repellent	371 (44.8)	333 (51.7)	38 (20.5)	< 0.001	4.14 (2.8-6.1)
Vitamins (e.g. vitamin C or multivitamins)	292 (35.2)	200 (31.1)	92 (49.7)	< 0.001	0.46 (0.3-0.6)
Anti-diarrhoeal medication (e.g. Imodium)	237 (28.6)	207 (32.1)	30 (16.2)	< 0.001	2.45 (1.6-3.7)
Antibiotics	184 (22.2)	149 (23.1)	35 (18.9)	0.2	-
Condoms	159 (19.2)	137 (21.3)	22 (11.9)	0.004	2.00 (1.2-3.2)
Anti-malarial medication	106 (12.8)	98 (15.2)	8 (4.3)	< 0.001	3.97 (1.9-8.3)
Vaccination card (with previous travel and non-travel vaccines)	90 (10.9)	76 (11.8)	14 (7.6)	0.1	-
Water purification kit	37 (4.5)	33 (5.1)	4 (2.2)	0.09	-
None of the above	47 (5.7)	24 (3.7)	23 (12.4)	< 0.001	0.27 (0.2-0.5)

* Pearson's Chi-square test

### Knowledge on travel diseases

One in five (19.8%, 330/1663) respondents incorrectly indicated the availability of a vaccine for the common cold, while 80.6% (1341/1663) were aware of the availability of an influenza vaccine. Respondents who had sought pre-travel advice from a health professional were more likely to be aware of the availability of vaccines for hepatitis A (OR: 1.4, 95% CI 1.0-1.9 *P *= 0.05), hepatitis B (OR: 1.8 95% CI 1.2-2.7 *P *= 0.002) and measles (OR: 1.8, 95% CI:1.2-2.6 *P *= 0.001). Compared to international students, domestic students were significantly more likely to be aware of the availability of vaccines for; hepatitis A (OR: 1.3 95%CI: 1.0-1.7 *P *= 0.03), hepatitis B (OR: 2.0, 95%CI: 1.5-2.5 *P *< 0.001), influenza (OR: 2.5 95%CI: 1.9-3.2 *P *< 0.001) and measles (OR: 3.7 95% CI: 2.9-4.8 *P *< 0.001).

### Risk perception

Overall, respondents did not feel overly worried about any of the listed travel threats, nor did they think they were 'highly likely' to occur while travelling abroad or in Australia. However, students did perceive that most of the threats were significantly more likely to occur while travelling overseas than in Australia. In contrast, respondents felt that a sexually transmitted infection (*p *< 0.001) and excessive sun exposure (*P *< 0.001) were more likely to occur while in Australia, while motor vehicle accidents were equally likely to occur in both setting (*P *= 0.3) (Table 3). Of the four infectious diseases rated, 40% of participants reported their health would be extremely affected by hepatitis A, 37.6% by dengue, 18.8% by measles and 9.3% by an influenza infection. In contrast to international students, domestic students were more likely to rate their health to be extremely affected if they contracted dengue (39.8% vs. 30% OR: 1.6, 95%CI: 1.2-2.0 *p *= 0.001) or hepatitis A (43.1% vs. 28.9%, OR: 1.9, 95%CI: 1.5-2.4 *P *< 0.0001). Students who nominated their health to be extremely affected by hepatitis A were 1.3 times more likely to report a previous vaccination for hepatitis A (OR: 1.3, 95%CI: 1.0-1.6 *P *= 0.03) and were more likely to have reported seeking pre-travel health advice from a health professional (OR: 1.5, 95%CI: 1.1-2.1 *P *= 0.005) and other sources (OR: 1.8, 95%CI: 1.2-2.5 *P *= 0.001). Students who reported their health would be extremely affected by dengue were also more likely to have sought advice from alternate sources other than health professionals (OR: 1.6, 95%CI: 1.1-2.2 *P *= 0.01).

**Table 3:** Mean likelihood and worry scores* for 12 health threats occurring while travelling internationally or in Australia, all student participants (N = 1663)

Travel threat	Likelihood			Worry		
	
	Travelling	Australia	***P *value**†	Travelling	Australia	***P *value**†
Illness from consuming contaminated food or water	3.18	1.84	< 0.001	2.95	1.82	< 0.001
Excessive sun exposure	3.17	3.58	< 0.001	2.48	2.88	< 0.001
Infection spread by coughing and sneezing (e.g. influenza, TB)	3.10	2.97	< 0.001	2.63	2.39	< 0.001
Robbery or assault	2.72	2.46	< 0.001	2.97	2.53	< 0.001
Infection transmitted through the bite of an insect (e.g. mosquito, tick)	2.66	1.92	< 0.001	2.47	1.85	< 0.001
Motor vehicle accident (e.g. as a pedestrian, bike, or in a vehicle)	2.56	2.58	0.30	2.62	2.56	0.02
Natural disaster (e.g. hurricane, flood, tsunami)	1.99	1.71	< 0.001	2.21	1.74	< 0.001
Dengue	1.86	1.42	< 0.001	1.87	1.51	< 0.001
Hepatitis A	1.72	1.51	< 0.001	1.83	1.57	< 0.001
Hepatitis B	1.71	1.52	< 0.001	1.86	1.60	< 0.001
Measles	1.58	1.49	< 0.001	1.66	1.49	< 0.001
Sexually transmitted infection (e.g. gonorrhoea, Chlamydia)	1.56	1.72	< 0.001	1.81	1.77	0.06

* mean score from a scale of 1 = not at all and 5 = very likely/worried

† *P *value for a paired samples t-test

## Discussion

Numerous surveys identify that travellers considerably underestimate the risks associated with travel to developing countries and a subsequent lack of preparation to avoid infectious disease risks [16-22]. This is especially true for younger travellers, who are disproportionately represented in studies assessing infectious symptoms post-travel [23-31]. For example, the incidence of post-travel illness in Swedish travellers who had attended a travel clinic for pre-travel health advice was greatest in those aged 10-24 years (65%) compared to those aged 25-49 years (49%) and those aged 50 years or more (33%) [23]. Younger travellers are at greater risk of infectious diseases which have been attributed to a higher susceptibility, particularly to vaccine-preventable diseases and greater exposure due to increased risk taking behaviours and travel to high-risk destinations [9,32]. Our study found a low risk perception across all of the health threats, for both domestic and international students, which support the previous findings of USA study abroad students [14]. Students in our study perceived a low personal likelihood of health threats occurring both in Australia and while travelling. The lowest likelihood and worry scores were for the individual infectious diseases included in the survey; dengue, hepatitis A, hepatitis B and measles, despite the fact that they are commonly reported in travellers [33]. Despite more than a third of students reporting that a hepatitis A or dengue infection would 'extremely affect' their health, the majority of students were barely worried. In a Finnish study of risk taking behaviours during travel, willingness to take health risks were associated with younger age [34], which may partially explain our results. Consistent low risk perceptions were also illustrated in a Canadian study on the beliefs and attitudes of students towards measles immunisations. In that study, 67% of students with no immunity to measles perceived little or no risk of acquiring measles and no need to be immunised for the disease [35].

In addition to low risk perception, was the low pre-travel preventative health preparation undertaken by our young, globally mobile student population. Uptake of pre-travel health advice was low overall with 68% of respondents reporting they had not sought any advice from a health professional prior to their last international trip. Our international students were far less likely to seek pre-travel advice from a health professional compared to their domestic counterparts. In the few studies that are available on student travellers, it is apparent that a large proportion of students do not seek health advice prior to travel [14,36]. For example, Abdullah et al reported that only 25% of Hong Kong students had sought pre-travel health advice, with 41% seeking information from 'non-expert' sources [36]. While it is likely that the level of pre-travel assessment and preparation will differ for students by destination (i.e. high vs low risk countries) and prior travel experience; travel consultation may still be required. For example, many young adult Australians are at risk of measles infection. In the past few years there have been several large and ongoing outbreaks of measles in a number of countries in Europe as well as in New Zealand and also several large outbreaks of mumps in the USA in educational institutions [37]. Given that many students are not aware of their immunisation status and many will travel during their university years (including multi-country trips to both high and low risk destinations), pre-travel consultation for this age group may increase awareness of destination-specific risks and provide protection for a lifetime of travel.

We also identified that there were variances amongst our participants in regards to the level of knowledge about travel-related diseases and their personal vaccination histories. Only one in two students were able to correctly identify the availability of the hepatitis A vaccine and more than one in five incorrectly reported that a vaccine for dengue existed. In addition, more than one in ten indicated that they did not know whether they had been previously vaccinated against measles, hepatitis A, hepatitis B and influenza. These deficits in knowledge are consistent with past studies of the general travelling population in which travellers are either unaware of their own immunisation status or have significant misimpressions of their vaccine history [10].

Gaps in knowledge of personal susceptibility and vaccination status may result in many travellers, including student travellers, choosing to forgo pre-travel vaccination assessments despite lack of existing immunity [10].

The reported uptake of vaccines was slightly higher amongst our participants than the rates reported by two previous studies. The first study by Hartjes et al. of USA study abroad students found that 42% had received at least one travel vaccine despite students studying in low income countries highly endemic for many vaccine-preventable diseases [14]. Lower uptake rates were reported in a Canadian study of tertiary education students, with 34.4% reporting a history of hepatitis A vaccination, despite 60% reporting prior travel and 81% reporting intention to travel to hepatitis A endemic countries [38]. Few travellers, including young adults, are able to accurately recall prior vaccination and information on vaccination coverage for university age Australians is not easily attainable. Australia has a vaccine registry but it is restricted to vaccines received prior to 7 years of age and to Australian's born during or since 1996. Australian children are registered on the Australian Childhood Immunisation Registrar (ACIR) upon registration with Medicare (99% of Australians) and can be added to the register by their health provider (including migrants) at anytime up to 7 years of age [39]. However, no register exists for older age groups in Australia and while there has been debate around the inclusion of a "whole-of-life" immunisation register, [40] no definitive actions have been undertaken. From 2013 onwards, Australian-born students or migrants (arriving prior to age 7) entering University in Australia will have been registered on the ACIR and vaccines received up to 7 years of age will be available to registered providers. In future cohorts of commencing university students, access to national vaccine registers may assist providers of travel health advice in determining missed childhood vaccinations and identifying at-risk young adults prior to travel. Other options for determining the vaccination status of international students is required.

Currently, there are no existing requirements at universities in Australia regarding proof of immunisation and status may only be reviewed if the student presents for a consultation. Education institutions may be the last opportunity to capture young adults in vaccination programs and universities clinics may provide the ideal opportunistic environment to improve vaccine coverage and thereby reduce travel risks. However, only 9 international and 15 domestic students indicated that they had attended the University Health Clinic, highlighting an under-utilised domain for travel health provision. Alternatively, education campaigns targeting young adults could also utilise the university networks and information gateways, such as distributing information brochures at the time of enrolment, during orientation week or through university-wide emails and newsletters. Other mediums favoured by young adults such as popular internet sites should also be considered as a possible means of information provision to this susceptible cohort and in increasing uptake of pre-travel preventative health advice. UNSW has embraced technology with campus information being delivered via Twitter and YouTube, while urgent SMS messaging, Facebook, Yammer and other similar sites could also be utilised. Given that the bulk of University community is under 25 years old it is an entirely appropriate to use this new technology.

Despite the university-wide broadcast of the survey and the obtained sample size being sufficiently large to draw statistical conclusions on the total UNSW population, the generalizability of our study is limited by the low response rate obtained. Our survey relied on a convenience sample, and as such may not be representative of the entire student population due to self-selection bias. However, information provided to students on the nature of the study prior to participation did not describe travel health or immunisation, but rather risk perceptions and practices towards infectious diseases in general and therefore may have reduced the opportunity for self-selecting by the students who have an interest in travel health or vaccination. Cross-sectional surveys, by their nature, are subject to recall bias. In this study, we asked students to describe their last episode of travel (undertaken in the previous year). Retrospective self-report of travel behaviours may have resulted in recall bias, contributing to the higher reported uptake rates.

## Conclusions

The population of domestic and international students to tertiary education institutions is large. In 2008, 1.1 million students were enrolled in public universities in Australia, including 770,000 domestic students [41]. Our study highlights the variability in the uptake of pre-travel health advice, vaccination and other precautions and the low risk perception in this age group. Limited data focus on young travellers despite high international student numbers and a high likelihood of travel in this age group. What we do know is that young adults, including university students, have a high likelihood of international travel. What we are not doing is targeting these students appropriately to ensure adequate protection from a lifetime of travel health risks. There is a need to improve health-seeking and knowledge of personal susceptibility and travel-health risks in this highly mobile young adult population. The next step is determining the most appropriate strategies for increasing pre-travel health preparation, and particularly for vaccine preventable diseases.

## Competing interests

CRM receives funding from influenza vaccine manufacturers GSK and CSL Biotherapies for investigator-driven research. These payments were not associated with this study. The remaining authors have no competing interests.

## Authors' contributions

MZ/HS/AH participated in the design of the study and survey, undertook the distribution and collection, performed the analysis and drafted the manuscript. CRM participated in the study design and coordination and in the editing of the manuscript. All authors read and approved the final manuscript.

## Pre-publication history

The pre-publication history for this paper can be accessed here:

http://www.biomedcentral.com/1471-2334/12/43/prepub
